# Uncomplicated Urinary Tract Infections and Antibiotic Resistance—Epidemiological and Mechanistic Aspects

**DOI:** 10.3390/antibiotics3030341

**Published:** 2014-07-22

**Authors:** Bernd Wiedemann, Anke Heisig, Peter Heisig

**Affiliations:** 1 Böstens Hoi 15, 24882 Schaalby, Germany; E-Mail: be-wiedemann@t-online.de; 2 Pharmaceutical Biology and Microbiology, Institute of Biochemistry and Molecular Biology, University of Hamburg, Bundesstrasse 45, 20146 Hamburg, Germany; E-Mail: anke.heisig@uni-hamburg.de

**Keywords:** uUTI, antibiotic resistance, fluoroquinolone, β-lactam

## Abstract

Uncomplicated urinary tract infections are typically monobacterial and are predominantly caused by *Escherichia coli*. Although several effective treatment options are available, the rates of antibiotic resistance in urinary isolates of *E. coli* have increased during the last decade. Knowledge of the actual local rates of antibiotic resistant pathogens as well as the underlying mechanisms are important factors in addition to the geographical location and the health state of the patient for choosing the most effective antibiotic treatment. Recommended treatment options include trimethoprim alone or in combination with sulfamethoxazol, fluoroquinolones, β-lactams, fosfomycin-trometamol, and nitrofurantoin. Three basic mechanisms of resistance to all antibiotics are known, *i.e.*, target alteration, reduced drug concentration and inactivation of the drug. These mechanisms—alone or in combination—contribute to resistance against the different antibiotic classes. With increasing prevalence, combinations of resistance mechanisms leading to multiple drug resistant (mdr) pathogens are being detected and have been associated with reduced fitness under *in vitro* situations. However, mdr clones among clinical isolates such as *E. coli* sequence type 131 (ST131) have successfully adapted in fitness and growth rate and are rapidly spreading as a worldwide predominating clone of extraintestinal pathogenic *E. coli*.

## 1. Introduction

Uncomplicated urinary tract infections are among the most common infectious diseases in the community and occur in patients without any anatomic or functional abnormality. About 50% to 70% of all women acquire such an infection at least once during their life [1]. Data on the prevalence and antibiotic resistance of the bacteria causing these infections are difficult to obtain, as these infections are treated empirically without bacteriological testing. However, the knowledge of the pathogens and their sensitivities towards the most commonly used antibiotics is essential for a successful treatment and helps avoiding development of resistance [2].

More than 90% of uncomplicated urinary tract infections are monomicrobial [3]. They are mainly (between 85% and 90%) caused by *E. coli*, to a lesser extent by other Enterobacteriaceae, Enterococci and Staphylococci [2]. The recent ECO.SENS study [4] reports a frequency of *E. coli* of 74.2% in patients from Austria, Greece, Portugal, Sweden, and UK. Similar frequencies for *E. coli* are reported by the ARESC study [5] and the findings by Dong Sup Lee *et al.* [6] In addition, these aforementioned studies report 3.4% and 2.3% *P. mirabilis*, 4.1% and 5.6% Enterococci, 3.5% and 4.7% *K. pneumoniae*, 1.1% and 2.3% *Enterobacter* spp., other bacteria were found with 11.2% and 7%, respectively. However, these data are derived from designed studies which have to be interpreted with caution: First, usually no microbiological testing is performed for patients suffering from uncomplicated urinary tract infections. Second, it is difficult to obtain reliable local data on the incidence of resistant strains from respective patients. Third, different surveillance systems do neither use the identical methodology to measure susceptibility nor the identical breakpoints for classifying resistant and sensitive bacteria. Beside laboratory data on the *in vitro* susceptibility of the presumptive causative agent, the application of antibiotic stewardship, which uses data not only on the local and global epidemiological situation of antibiotic resistance, but also the potential impact of antibiotics on the microflora of the patient, provides a rationale for choosing an appropriate antibiotic for treatment. The aim of this review is to provide an overview of epidemiology and mechanisms of resistance for antibiotics frequently used in the treatment of uncomplicated urinary tract infections.

## 2. Therapeutic Options

Besides pharmacodynamic, pharmacokinetic, and tolerability aspects developing or already existing resistance to the drug to be chosen for treatment is the most important motivation for selecting an antibiotic. Furthermore, the activity of the drug against the resident bowel flora, as well as the effect of the duration of the treatment on the probability to develop resistant bacteria may have an important impact [1].

Considering the afore mentioned factors, therapeutic options for the treatment of uncomplicated, community acquired urinary tract infections have been developed, based on few long experienced antibiotics for oral application. The treatment of acute uncomplicated cystitis as recommended by the guidelines of the European Association of Urology (EAU) [7] includes fosfomycin-tromethamol, pivmecillinam, and nitrofurantoin as first line therapy. As an alternative therapy, fluoroquinolones, cefpodoxime proxetil, cotrimoxazole and trimethoprim are possible options, if the local resistance rate is less than 20%. These recommendations should be adjusted taking into account the geographical location of the patient, age, and sex as well as other diseases. A similar restriction is valid for β-lactam antibiotics like amoxicillin, amoxicillin/clavulanic acid and pivmecillinam, which are used in some countries. The recommended duration of treatment takes into account the necessary time for effectiveness and the risk of resistance development as a result of prolonged selective pressure. Usually, the duration of treatment is three days. Fosfomycin is given even in a single dose, amoxicillin and nitrofurantoin, however, require five to seven-day treatment [8].

## 3. Antibiotic Resistance—Genetic and Mechanistic Basis

The development of antibiotic resistant mutants from susceptible cells is driven by two characteristic features of prokaryotic cells—high growth rate and a haploid genome: The rapid growth rate yields a large population size within a short period of time and this increases the probability to yield one mutant cell within typically 10^8^ cells due to the fortuitous acquisition of a resistance mutation [9]. Slow growing bacteria, like *Mycobacterium tuberculosis* overcome their growth deficiency by expression of immune evasion mechanisms which ensure the undisturbed local persistence of populations over periods of time long enough to develop resistant mutants [10].

The haploid genome structure allows for an immediate expression of a mutant genotype in bacteria. Beside mutations, which alter existing genetic material resulting in antibiotic resistance, another genetic strategy to acquire antibiotic resistance is the transfer of genetic material encoding antibiotic resistance between cells of mixed bacterial populations by either transformation of naked DNA, conjugation of plasmid DNA via cell-cell-contact, or phage-mediated transduction [11]. Either genetic alteration can result in one of three basic biochemical mechanisms of resistance: a reduction of the affinity of the target for an antibiotic, a reduction of the concentration of the drug at the target site, and an enzymatic inactivation of the drug. During evolution under selective pressure, bacterial cells have developed numerous variations of these three basic mechanisms which alone or in combination can result in clinically relevant resistance to specific or even all known antibiotics [12]. However, the acquisition of antibiotic resistance can be associated with a reduced fitness/virulence of the resistant cell due to an impairment of the normal function of the affected target or to the overexpression of a specific resistance gene [13,14]. While in the presence of the selecting antibiotic the resistant mutant has a growth advantage, in the absence of the antibiotic, *i.e.*, after the therapy is completed, reduced fitness can turn this into a disadvantage. Over time, antibiotic is resistant, but less fit mutants can acquire additional genetic alterations which enable them to compensate for the fitness reduction. Finally, the combination of resistance and compensatory mutations can give rise to well adapted clones capable of spreading among host populations.

## 4. Resistance to Sulfonamides and Trimethoprim—Epidemiology and Mechanisms

The use of sulfonamides alone or in combination with the dihydropyrimidine derivative trimethoprim has a long history in the treatment of uncomplicated urinary tract infections. Since these infections are often treated empirically without susceptibility testing only a few data on resistance of the pathogen are available. Percentages of *E. coli* strains resistant to cotrimoxazole vary with the geographical location of the patients: 35.9% in Korea [6], 25.4% [15], 16.1% [4], and 12.2% [16] in Europe. According to recent data from Kahlmeter *et al.* [4] the percentages are 15.9, 18.2, 16.7, 16.3, and 14.4 for Austria, Greece, Portugal, Sweden and UK, respectively. These authors also described changing incidences from the first study in 1999–2000 to the second study in 2008: Portugal showed a drop from 26.7% to 16.7% and Sweden an increase from 8.3% to 16.3% resistant strains. Data for sulfonamides and trimethoprim alone are scarce, 24.8% and 16.7%, respectively [6].

Sulfonamides such as sulfamethoxazol (SMX) and dihydropyrimidines such as trimethoprim (TMP) target dihydropteroate synthetase (DHPS, the *sul* gene product) and dihydrofolate reductase (DHFR, the *dfr* gene product), respectively. Both enzymes catalyze either of two subsequent steps in the bacterial biosynthesis of folic acid [17]. Recent crystallographic data revealed that a conserved binding pocket of DHPS for the natural substrate para-aminobenzoic acid (p-ABA) is formed only in an intermediate reaction step during the catalytic cycle. The resulting covalent bond formed between p-ABA and a pteridin cation yields 7,8-dihydropteroate [18]. In an analogous mode of action involving dynamic conformational changes of DHFR from a closed to an occluded state, this enzyme catalyzes the reduction of the substrate dihydrofolate to tetrahydrofolate (THF) by using nicotinamide adenine dinucleotide phosphate (NADPH) as a cofactor [19]. Inhibition of either enzyme by sulfonamide or trimethoprim causes a shortage of folic acid, which is an essential cofactor also for the biosynthesis of purine nucleotides, thymine, nucleic acids, and serine. As a consequence DNA replication stops and this event finally causes cell death.

Resistance to SMX and TMP could easily be selected *in vitro* from susceptible strains of *E. coli*. Resulting mutants were demonstrated to have acquired mutational alterations of the chromosomal *sul* and *dfr* genes. Such modifications have also been identified as a cause of primary resistance to sulfonamide in chromosomal *sul* genes of those species which are naturally competent pathogens such as *Streptococcus pneumoniae*, *Campylobacter jejuni*, *Neisseria meningitidis*, and *Neisseria gonorrhoeae*, capable of taking up from the environment foreign DNA fragments released from dead cells, and subsequently integrating them into their chromosome. However, comparison of *sul* and *dfr* gene sequences from isolates belonging to the above mentioned species, suggest a horizontal transfer of antibiotic resistant gene copies and subsequent integration of resistance-determining gene sequences from the acquired gene into homologous regions of the chromosomal copy. The resulting sequences form so-called mosaic genes carrying a central antibiotic resistant gene fragment of acquired DNA flanked by regions of the residing chromosomal gene copy [20].

In contrast, the most frequent mechanism of resistance in clinical isolates of *E. coli* and other enterobacteria from urinary tract infections is the acquisition of resistant variants of complete *sul* and *dfr* genes expressing enzyme variants which are refractory to the inhibitory activity of the respective drug at clinically achievable concentrations. More than 30 resistant variants of trimethoprim resistant *dfr* genes and three variants of sulfonamide resistant *sul* genes have been described so far [20]. Many of these are encoded by mobile genetic elements residing on transferable plasmids in combination with other resistance genes. As a consequence, multiple resistance genes are cotransferred en bloc. An unusual genetic constellation has been detected in sulfonamide resistant *E. coli* isolates belonging to clonal group A, which have been isolated from different US regions. All isolates carry a genomic resistance module consisting of several resistance genes integrated in a specific chromosomal locus [21].

## 5. Resistance to Fluoroquinolones—Epidemiology and Mechanisms

Resistance to fluoroquinolones in *E. coli* is quite high in many European countries ranging from 25% to 50% [22]. The prevalence of resistant strains, according to few surveillance data available on uncomplicated urinary tract infections, was reported to be much lower, *i.e.*, 28.2%, 13.9%, 3.9%, and 1.0% according to references [4,6,15,16], respectively. However, again Kahlmeter, 2012 [4] reports an increase from 2000 to 2008 in most countries in Austria (0% to 4.1%), in Greece (1.5% to 5.7%), in Portugal (5.8% to 7.6%), in Sweden (0% to 2.5%), and in the United Kingdom (0.5% to 0.6%). The incidence of resistant strains isolated from blood in 2008 was 22.9% in Austria, 22.4% in Greece, 28.6% in Portugal, 10.3% in Sweden, and 15.1% in the United Kingdom according to EARS Net. The source of infection in uncomplicated UTIs is the bowel flora of the patient or the sexual partner. The resistance is much lower in relation to surveillance data from hospitals.

Fluoroquinolones were first introduced into clinical use in 1985. The high clinical efficacy of orally available fluoroquinolones norfloxacin, ofloxacin and ciprofloxacin together with the initially very low incidence of resistance in *E. coli* and many other Gram-negative pathogens in the treatment of urinary tract infections rapidly resulted in a widespread empirical use for this application. This high efficacy is due to the high affinity and inhibitory activity of the drugs to their target topoisomerases gyrase and topoisomerase IV which are A_2_B_2_ tetrameric enzymes sharing high structural and functional homology. Consequences of the irreversible enzyme inhibition are the arrest of replicative DNA metabolism and subsequent cell death due to secondary bactericidal mechanisms such as the introduction of DNA double strand breaks following the inhibition of DNA gyrase [23,24].

The development of clinically relevant resistance to fluoroquinolones in *E. coli* has been investigated intensively and has been demonstrated to require multiple mutation steps which involve alterations in conserved regions of both chromosomally encoded target gene pairs *gyrA*/*parC* and *gyrB*/*parE* encoding subunits A and B of topoisomerase II/IV, respectively. These result in a reduced affinity of the drugs to the mutated target [25]. In addition, a reduced drug concentration at the target site had been associated with chromosomal mutations either reducing the amount of outer membrane porin OmpF, a water-filled transmembrane channel which allows water-soluble small molecules such as fluoroquinolones to enter the cell, or by an increased expression of multiple drug-resistance (MDR) efflux pump AcrAB-TolC which actively exports antibiotics of different classes out of the cell, or by a combination of both. The latter mechanism is due to genetic alterations inactivating chromosomally encoded local (AcrR) or global negative regulators (MarR, SoxR, and RamR) which control the expression of MDR efflux pump AcrAB-TolC. Global regulators have been demonstrated to simultaneously control the expression of major porin OmpF via an antisense RNA switch.

Besides these mechanisms, several non-target based mechanisms of plasmid-mediated quinolone resistance (PMQR) have been detected during the last decade. A PMQR mechanism alone mediates only a low-level fluoroquinolone resistance resulting in MIC increases below the breakpoint. PMQR are subdivided into (I) mechanisms associated with reduced drug concentration at the target site due to the expression of plasmid-encoded quinolone efflux pumps QepA or OqxAB, (II) mechanisms protecting the target site, as has been determined for the different Qnr proteins belonging to either of the pentapeptid repeat protein families QnrA, QnrB, QnrC, QnrD, or QnrS, and (III) a mechanism exclusively inactivating C7-piperazinyl substituted fluoroquinolones norfloxacin and ciprofloxacin by an acetyltransferase mechanism. This unique enzyme is derived from an aminoglycoside-modifying acetyltransferase AAC6’(Ib) by the acquisition of two point mutations which extend the substrate spectrum to two different antibiotic classes [26].

While mechanisms of PMQR are reported with increasing prevalence also in *E. coli* isolates mediating UTI in humans and animals, their impact on clinically relevant resistance is lower compared to mutations affecting target topoisomerases gyrase and topoisomerase IV, but in combination PMQR contribute to an increase in the resistance level [27]. In addition, a possible role of PMQR as pacemakers of the development of clinical resistance to fluoroquinolones is being discussed. This view is supported by *in vitro* studies demonstrating an impact of *qnr* genes in *E. coli* isolates from UTI on fluoroquinolone activity in an *in vitro* model [28] as well as in a mouse infection model [29,30]. Former use of fluoroquinolones has been identified as a relevant risk factor for the development of clinically relevant resistance to these drugs [31].

## 6. Mechanisms of Resistance to β-Lactam Antibiotics—Epidemiology and Mechanisms

For uUTI treatment pivmecillinam (PIV), the prodrug of the active compound mecillinam, is recommended as first-line drug in several countries [1]. PIV shows good clinical cure rates against Gram-negative pathogens including *E. coli* expressing extended-spectrum β-lactamases (ESBL) such as ST131 isolates encoding CTX-M14, CTX-M15 [32]. Therefore, resistance to cefotaxime or ceftazidime can be used as a good marker for ESBL prevalence. Such data obtained from EcoSens study II revealed increasing but still low prevalence of ESBL producing *E. coli* from uUTI in Europe [4]. Due to high concentrations of β-lactam drugs in the bowel and a relatively long treatment period all β-lactams exert a high degree of selection pressure. Prevalence of resistant *E. coli* strains for amoxicillin is far above 20%, so that this drug should not be used for treatment of these infections at all. The addition of clavulanic acid restores susceptibility for most strains. Data from Kahlmeter [4] demonstrate that the percentage of amoxicillin/clavulanic acid-resistant *E. coli* strains differs from country to country: Austria, 8.9%, Greece 4.3%, Portugal 6.9%, Sweden 2.5%, and United Kingdom 2.0%. Data from other studies also show a varying realation of *E. coli* isolates from uncomplicated urinary tract infections resistant to amoxicillin/resistant to amoxicillin plus clavulanic acid, such as 63.6%/5.5% [6], 42.4/7.5 [15], 28.0/4.5 [4], and 35.5/1.5 [16].

The predominant mechanism of resistance to β-lactam antibiotics in *E. coli* and most other Gram-negatives is the production of a β-lactamase which enzymatically inactivates β-lactam antibiotics by hydrolysis of the essential β-lactam ring. As a consequence the β-lactam will not more bind to its targets, the cell wall synthesizing transpeptidases/transglycosylases also designated as penicillin binding proteins (PBPs). According to the active site architecture β-lactamases either belong to the serine protease type or to the metallo enzyme type. While the catalytic site of a serine protease is formed by a conserved triad of amino acids aspartate, histidine and serine [33], that of a metallo enzyme is composed of a central catalytically active Zn^2+^ ion chelated by a set of four conserved histidin and/or cystein amino acids [34]. While some enterobacterial genera encode a chromosomal β-lactamase active against a broad spectrum of β-lactam antibiotics whose expression can be induced by β-lactams, the clinically most relevant enzymes are plasmid-encoded extended spectrum β-lactamases (ESBL), serine β-lactamases, which hydrolyze most β-lactams with the exception of carbapenems, but can be inhibited by β-lactamase inhibitors such as clavulanic acid [35].

Many ESBL enzymes belonging to class A are grouped into one of three major families: TEM, SHV and CTX-M. Within each family a high degree of DNA and amino acid sequence homology is found. Individual family members differ from their parent enzyme mediating only broad-spectrum activity by a few point mutations. These mutations affect regions associated either with the access of the drug to the binding pocket containing the active-site serine or with the kinetic properties of the enzyme resulting in an acceleration of drug inactivation. The resulting enzymes mediate resistance to extended spectrum β-lactams which includes resistance to cefotaxime and ceftazidime as a good marker for an ESBL phenotype [36]. Beside these “classical” ESBL enzymes, several new derivatives of classes C and D β-lactamases have evolved which share characteristics of an ESBL phenotype [37]. Despite of an increasing prevalence of ESBL-producing *E. coli* in UTI the clinical efficacy of β-lactams in the treatment of acute uncomplicated urinaryt tract infections is often less affected by this resistance mechanism presumably due to the high drug concentrations in urine [32]. *E. coli* strains belonging to the epidemiologically dominating sequence type 131 (ST131) express a multiple drug resistance phenotype which includes sulfonamide/trimethoprime and fluoroquinolones in addition to β-lactams due to the acquisition of a plasmid carrying a *bla_CTX-M-15_*-encoding ESBL gene [37].

## 7. Resistance to Fosfomycin—Epidemiology and Mechanisms

Fosfomycin is rarely used in clinical settings, it is used only in combination with other powerful drugs like third generation cephalosporins, carbapenems, or aminoglycosides in severe infections, because the mutation rate to generate resistant mutants is extremely high. For uncomplicated urinary tract infections, however, the monobasic watersoluble fosfomycin salt, fosfomycin trometamol, is specially designed. After oral application, fosfomycin achieves a urinary concentration, which does not allow the resistant mutants to grow. Therefore, it can be used in this indication as a single drug.

Until now, resistance rates to fosfomycin are low: 1.2% and 0.2% acording to [4] and [16], respectively. Kahlmeter [4] found a low prevalence of fosfomycin resistance for all European countries involved, such as Austria 0.7%, Greece 2.9%, Portugal 0.7%, Sweden 1.5%, and United Kingdom 1.5%. A systematic review of clinical studies on the incidence of fosfomycin resistance in clinical isolates from complicated and uncomplicated urinary tract infections shows less than 8% resistance overall [38].

Fosfomycin is a potent inhibitor of the *murA* gene product, UDP-N-acetylglucosamine enoylpyruvyl transferase, which catalyzes an essential step in the synthesis of UDP-N-acetylmuramic acid, one essential building block for peptidoglycan synthesis. Inhibition of MurA results in an effective stop of the biosynthesis of the bacterial cell wall. However, to inhibit the intracellular target structure MurA, fosfomycin has to pass the cell membrane. This is achieved in *E. coli* by an active uptake process involving either one of two fosfomycin influx transporters, gylcerol-3-phosphate transporter (the *glpT* gene product) and hexose phosphate transporter (the *uhpT* gene product) [39]. Different mechanisms of resistance to fosfomycin have been identified from *in vitro* studies. These mechanisms include in *E. coli* a point mutation in *murA* changing cys-115 into asp thereby preventing the covalent binding of fosfomycin to its target [40]. Variations at this site have been detected in species such as *M. tuberculosis*, *Chlamydia trachomatis*, and *Vibrio fisheri* which are naturally less susceptible to fosfomycin. Another mechanism involves mutations in genes *uhpT* and *glpT* which results in reduced drug uptake [39]. However, these chromosomal mutations selected *in vitro* are associated with reduced fitness. This seems to provide a plausible explanation for the observed low incidence of such mutants in clinical isolates from urinary tract infections in comparison with the relatively frequent isolation of resistant mutants *in vitro* [41]. However, recent data report the occurrence of clinical isolates in Asia with transferable resistance to fosfomycin. The underlying mechanism is the expression of a glutathione-S-transferase activity which inactivates fosfomycin. Several genes encoding such activity have been identified on resistance plasmids frequently associated with a gene encoding a CTX-M-type β-lactamase [42,43].

## 8. Resistance to Nitrofurantoin—Epidemiology and Mechanisms

Nitrofurantoin is the class representative of the nitroimidazoles. After intracellular activation by bacterial nitroreductases it shows excellent bactericidal activity against *E. coli* and a still good, but lesser activity against many other enterobacterial pathogens. This advantage of an as yet extraordinarily low incidence of resistance (<3%) is partially outcompeted by an increased risk for severe side effects, such as toxicity for lung and liver [44].

## 9. Epidemiological Aspects of Multiple Drug Resistance in Urinary Tract Infections

During the last decade the isolation of ESBL-producing *E. coli* isolates belonging to the O25b:H4 serotype from urinary tract infections has increasingly been reported from over the world. Detailed molecular research revealed that these isolates predominantly belong to ST131 and the pathotype B2. Remarkable is the high incidence of fluoroquinolone resistance due to chromosomal mutations in both target topoisomerases [45] as well as the presence of a conjugative plasmid typically carrying a CTX-M-type β-lactamase such as −15, −1, and −14 [46,47]. A genome analysis of a set of ST131 isolates provided further evidence for a single clone which has spread among patients suffering from community acquired urinary tract infections within a large UK region and has split into a few molecular subgroups presumably due to the acquisition of resistance plasmids mediating varying patterns of antibiotic resistance including β-lactams, trimethoprim/sulfonamide, tetracycline, or gentamicin [48]. Besides a resistance profile the ST131 cells seem to express specific virulence traits allowing them to successfully spread among patients not only in hospitals, but also in nursing-homes. The detection of ST131 cells in animals varies between different studies suggesting that animals do not play a significant role as reservoirs for human infections. Although local epidemiological data from the Indian subcontinent are limited [49], travel to Pakistan and India is suspected to be a potential risk factor for the acquisition of multiple drug resistant *E. coli* ST131 [47]. Thus, the epidemiological survey of this pandemic clone requires special attention in the future and may have an impact on the empirical treatment of uncomplicated urinary tract infections.
